# Polylactic Acid Membranes, a Novel Adjunct Treatment for Bullous Impetigo

**DOI:** 10.3390/idr17030072

**Published:** 2025-06-19

**Authors:** Ana Lorena Novoa-Moreno, Mario Aurelio Martinez-Jimenez, Arturo Ortiz-Alvarez, Natalia Sanchez-Olivo, Victor Manuel Loza-Gonzalez, Jose Luis Ramirez-GarciaLuna

**Affiliations:** 1 Wound Clinic of the Regional High-Specialty Hospital “Ignacio Morones Prieto”, San Luis Potosí 78220, SLP, Mexicomario.jimenez@uaslp.mx (M.A.M.-J.); vic.loza.gonzalez@gmail.com (V.M.L.-G.); 2 Infectology Department of the Regional High-Specialty Hospital “Ignacio Morones Prieto”, San Luis Potosí 78220, SLP, Mexico; 3 Division of Experimental Surgery, Faculty of Medicine, McGill University, Montreal, QC H3G 1A4, Canada

**Keywords:** skin infections, impetigo, polylactic-acid-based biomaterials, wound healing, local anti-infective agents

## Abstract

Impetigo is a highly contagious bacterial skin infection characterized by blistering and erosions that can lead to significant discomfort and complications. The standard treatment includes topical or systemic antibiotics, but severe cases may require advanced wound management strategies. Polylactic acid (PLA)-based membranes have demonstrated effectiveness in enhancing wound healing, modulating inflammation, and reducing pain. **Clinical case:** We present three cases of bullous impetigo with extensive erosions, managed using PLA membranes as an adjunct to systemic antibiotics. A significant improvement was shown after 7 days of treatment of a single application, and complete resolution was achieved after 30 days. Notably, pain was resolved within 48–72 h, highlighting the analgesic and protective properties of the membrane. **Conclusions:** These findings suggest that PLA membranes provide a viable adjunct to antibiotic therapy in bullous impetigo, accelerating healing, reducing discomfort, and improving long-term skin outcomes. Given the increasing concern over antibiotic resistance and the limitations of standard wound care, bioresorbable synthetic membranes represent a promising alternative in dermatological wound management.

## 1. Introduction

Impetigo is a common and highly contagious bacterial infection of the skin, most often seen in young children, although it can occur in all age groups. Clinically, it is classified into non-bullous (70% of cases) and bullous forms (30%), each with distinct pathophysiology and microbiological profiles [1,2]. Non-bullous impetigo, the most prevalent form, is primarily associated with *Streptococcus pyogenes* and *Staphylococcus aureus*. In contrast, bullous impetigo is exclusively mediated by *S. aureus*, which produces exfoliative toxins (ETA and ETB), leading to the formation of intraepidermal blisters via desmoglein-1 cleavage [3,4]. Lesions frequently arise in exposed areas, often following minor cutaneous trauma, insect bites, or pre-existing dermatological conditions such as atopic or seborrheic dermatitis. While typically self-limiting, complications can extend beyond localized infection, with ecthyma representing a deeper ulcerative progression, extensive blisters that are exquisitely painful, scalded skin syndrome, and, in rare instances, post-streptococcal glomerulonephritis, posing a systemic risk in *S. pyogenes*-associated cases [5].

The current management relies on topical or systemic antibiotics, with mupirocin, fusidic acid, and oral β-lactams being the standard first-line options. However, the increasing global prevalence of methicillin-resistant *S. aureus* (MRSA) and emerging resistance to fusidic acid have underscored the need for alternative therapeutic strategies that mitigate antimicrobial overuse while effectively controlling the bacterial burden [6,7]. One such alternative therapy is Suprathel^®^ (Polymedics Innovations, Kirchheim unter Teck, Germany), a polylactic acid (PLA)-based alloplastic skin substitute. This biomaterial has shown excellent outcomes in bacterial load reduction [8], modulation of the wound microenvironment, and enhanced re-epithelialization in partial-thickness burns [9,10], toxic epidermal necrolysis (TEN) [11,12], and epidermolysis bullosa [13], thereby offering a potential adjunct to the conventional antibiotic therapies in superficial bacterial skin infections.

Suprathel^®^ is a fully synthetic resorbable skin substitute composed of 75% polylactic acid and 25% other copolymers, designed to serve as a temporary epidermal barrier in partial-thickness wounds, burns, and skin graft donor sites. Its unique semi-permeable structure allows for the optimal moisture retention, oxygen permeability, and controlled exudate management, creating a wound environment that supports re-epithelialization and pain reduction [14]. One of its key advantages is its ability to closely mimic the properties of the epidermis, providing mechanical protection while promoting keratinocyte migration and proliferation. Upon application, the membrane adheres to the wound bed without requiring fixation and is designed to degrade spontaneously and gradually through hydrolysis of its ester bonds. This process releases lactate/lactic acid monomers as its primary byproduct, further modulating the wound microenvironment and reducing the local pH [15]. This acidic environment indirectly acts as a natural antimicrobial mechanism in which common wound pathogens such as *S. aureus* and *Pseudomonas aeruginosa* have low survival and proliferation capacities [16].

Given our group’s experience utilizing Suprathel^®^ in the treatment of burns and chronic wounds, combined with its well-documented properties, we propose that this product could serve as an innovative approach to managing impetigo-related wounds, particularly in cases where extensive skin loss, superinfection, or chronicity poses challenges to standard antibiotic-based treatments. Its potential to modulate the wound microenvironment, reduce the bacterial load, and enhance epithelial regeneration aligns with the emerging need for alternative, non-antibiotic wound therapies, particularly in an era of increasing antibiotic resistance.

## 2. The Case Description

We present the cases of three female patients, aged 50 to 90 years, who developed bullous impetigo of the lower extremities. In all cases, the condition initially manifested as fluid-filled blisters, which progressively coalesced into extensive, painful erosions covered with amber-colored crusts. Clinical examination revealed signs of an advanced local inflammatory response, including extensive erythema, edema, warmth, and tenderness, with or without regional lymphadenopathy. Microbiological cultures confirmed *S. aureus* in all patients, and systemic antibiotic therapy was initiated following the Surgical Infection Society Guidelines for managing complicated skin and soft tissue infections [17].

As an adjunct to oral antibiotic therapy, Suprathel^®^ membranes were applied to the wounds. Following the manufacturer guidelines, the membranes were placed in direct contact with the wound bed, followed by a non-adherent paraffin gauze layer to prevent displacement. An outer absorbent dressing and a mild compressive bandage were applied to protect the membranes and optimize the wound healing conditions.

As a control group, we included five patients aged 60 to 82 years old with similar infections that were treated using systemic antibiotics and mupirocin ointment as an adjunct to care. Mupirocin was instructed to be applied daily, twice per day, and the wounds covered with sterile gauzes. A summary of the patients’ demographic and clinical characteristics is presented in Table 1.

Patients were managed entirely in the outpatient setting and instructed to return for weekly dressing changes until complete re-epithelialization was achieved. At each visit, the dressings were carefully removed, and the healing progress and the integrity of the tissue were assessed. For the patients that received Suprathel^®^, the membranes were also evaluated. Detached Suprathel^®^ fragments and hyperkeratotic crusts were selectively debrided as needed. Due to the membrane’s translucency upon application, a progressive improvement in the underlying skin was visible throughout the treatment course. Complete wound healing was observed in all cases, with spontaneous detachment of the Suprathel^®^ membranes marking full epithelialization. Patients were discharged from clinical follow-up on day 30 and advised to continue post-treatment care with emollient oil to maintain skin hydration, optimize epidermal recovery, and minimize recurrence risk.

In contrast, none of the patients treated with topical mupirocin achieved full closure within the 12-week follow-up period. Four of the five patients experienced partial healing by day 21 but later developed complications, including severe hyperkeratosis, impetigo recurrence, and secondary bacterial infections.

## 3. Results

Before applying the PLA membrane, all patients reported moderate to severe pain associated with their wounds, characterized by persistent discomfort, tenderness, and hypersensitivity to touch. Within hours of the membrane’s application, the patients noted a significant reduction in pain intensity, and by day 2–3, complete pain relief was reported in all cases. No additional analgesia was required beyond the standard wound care measures, suggesting that the PLA matrix provided an effective barrier function while modulating the inflammatory response.

**Figure 1:**
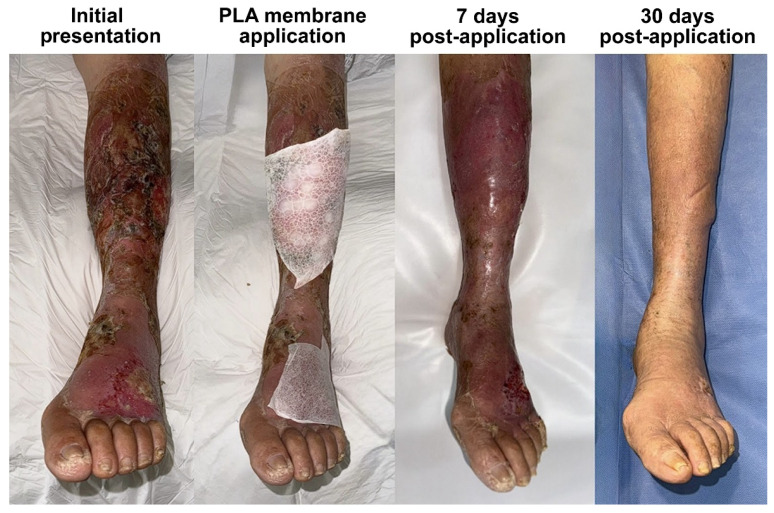
The clinical course of bullous impetigo in a 61-year-old patient with diabetes, presenting with lesions on the dorsum of the left foot and the anterior aspect of the leg, who was hospitalized previously due to a septic shock and presented to our charge once discharged. Each of the affected areas were treated using a PLA membrane, leading to complete epithelialization within 7 days and full restoration of normal skin characteristics by day 30. This case illustrates the ability of PLA membranes to enhance the final tissue quality after healing.

**Figure 2:**
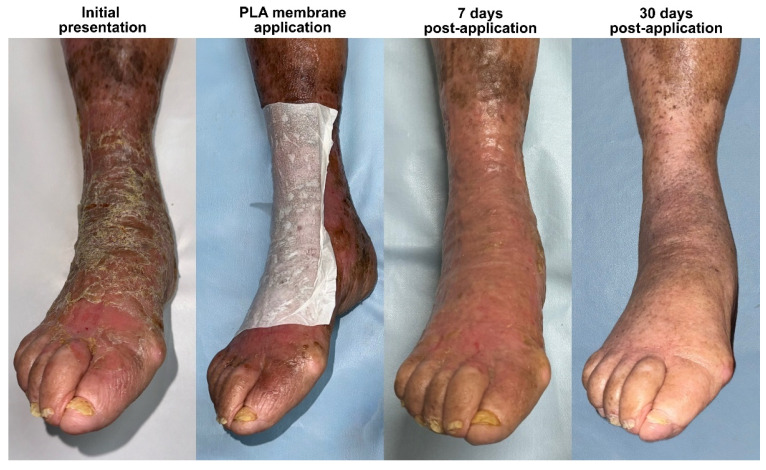
Bullous impetigo following an insect bite in a 90-year-old patient with mild chronic venous insufficiency. The infection led to extensive skin desquamation, worsened by mechanical irritation from scratching. Following debridement and the application of a PLA membrane, the patient experienced rapid relief of pain and pruritus, highlighting the membrane’s role in symptom control and epidermal protection. Re-epithelialization was achieved within 7 days, and by day 30, the tissue had fully remodeled, exhibiting a smoother and healthier appearance compared to that of the surrounding skin, which still displayed mild venous stasis-related changes. This case highlights the efficacy of PLA-based membranes in a frail, elderly, and potentially immunocompromised patient, demonstrating their ability to accelerate wound healing, modulate the inflammatory response, and promote scar-free tissue regeneration despite the impaired skin repair mechanisms commonly associated with aging and diabetes.

**Figure 3:**
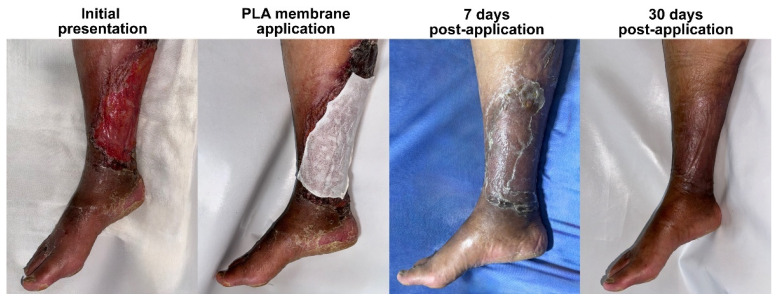
The clinical progression of bullous impetigo in a 50-year-old female patient with C4 chronic venous insufficiency. Due to pre-existing venous stasis and poor tissue quality, the lesions progressed rapidly, resulting in an extensive lesion affecting the medial and posterior aspects of the right leg. Treatment with a PLA membrane led to full re-epithelialization within 14 days, with the initial tissue deficit likely contributing to the delayed healing compared to that in other cases. The 7-day image shows adhered translucent Suprathel^®^, indicating that epithelialization is incomplete, and reinforcing the importance of leaving the membrane undisturbed until spontaneous detachment occurs. By day 30, the skin structure was fully restored, with persistent hyperpigmentation due to underlying venous insufficiency, but no residual inflammation, scarring, or recurrent ulceration. This case underscores the effectiveness of PLA-based membranes in challenging wound environments, demonstrating their ability to support dermal repair despite vascular compromise.

In addition to pain relief, rapid and sustained re-epithelialization was observed in all patients. Two patients achieved full epidermal coverage within 7 days (Figure 1 and Figure 2), while the third patient, whose lesions were more extensive and deeper at the baseline, achieved complete epithelialization by day 14 (Figure 3). Throughout the healing process, there was no evidence of lesion progression, new blister formation, or secondary infections, indicating that the membrane provided an effective protective microenvironment for uninterrupted tissue regeneration.

At each follow-up visit, the integrity of the Suprathel^®^ membrane was assessed, revealing progressive thinning and translucency in the non-epithelized areas of the wound, which allowed for direct visualization of the quality of the underlying tissue. In the patients who healed within the first week, the residual membrane appeared yellowish and crusty and could be removed through gentle scraping with gauze.

By day 30, all patients exhibited fully remodeled skin with no residual inflammation, erythema, or scarring. The healed areas were indistinguishable from the surrounding unaffected skin, demonstrating that PLA-membrane-assisted wound closure facilitated the optimal dermal restructuring. Notably, except for a patient with previous trophic changes due to chronic venous insufficiency in the leg, there was no evidence of post-inflammatory hyperpigmentation or hypertrophic scarring, which are common concerns in post-bullous-impetigo wound healing. After healing, the patients reported no discomfort, pruritus, or residual tenderness, further supporting the notion that PLA-based wound therapy promotes restoration of the epidermal barrier function without excessive fibrotic remodeling.

Importantly, all patients were treated exclusively in an outpatient setting, requiring only weekly dressing changes. The lack of complications, the absence of secondary bacterial superinfections, and the accelerated wound healing timeline collectively highlight the potential of Suprathel^®^ as a viable alternative to the traditional wound dressings in impetigo-associated skin erosions. The early establishment of an intact epidermal barrier not only minimized the infection risk but also eliminated the need for prolonged wound management interventions, thereby reducing the patients’ burden and healthcare resource utilization.

**Figure 4:**
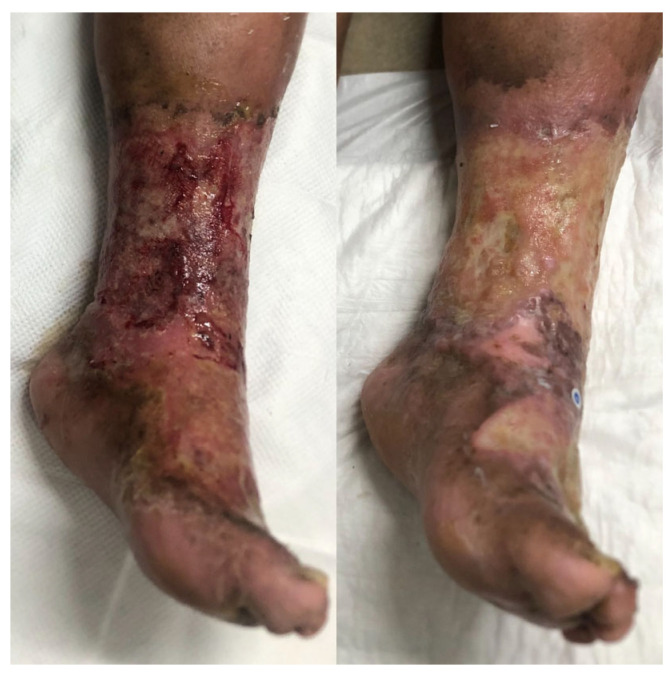
The clinical evolution of a 69-year-old female patient with bullous impetigo and a history of diabetes mellitus type 2 in poor-control, chronic venous insufficiency and active heavy smoking. The left panel shows the initial presentation. Oral antibiotic treatment was initiated (amoxicillin–clavulanate and trimethoprim–sulfamethoxazole), and topical mupirocin was used for 14 days. After 21 days of evolution (right panel), the wound worsened, and a superinfection with *E. coli* was diagnosed. The patient continued wound care for a total of 12 weeks, during which she presented with several recurrences and tissue breakdown.

All patients in the control group were monitored over a 12-week period. Despite treatment, none achieved full wound closure. Three patients exhibited partial epithelialization by day 21, yet all experienced recurrence of tissue breakdown during the follow-up. Notably, extensive inflammatory hyperkeratosis developed in three individuals, and one case was further complicated by a bacterial superinfection with *E. coli* (Figure 4). These findings underscore the limitations of the conventional topical antimicrobial strategies in achieving durable resolution in bullous impetigo, particularly in patients with underlying comorbidities such as heavy smoking, diabetes mellitus, and chronic venous insufficiency.

Taken together, these findings suggest that the application of PLA membranes in bullous impetigo lesions offers a multifaceted therapeutic advantage, including adequate pain control, rapid re-epithelialization, prevention of lesion progression, and scar-free remodeling. These results support further investigations into the role of bioresorbable synthetic membranes in dermatological wound management, particularly in the context of non-surgical alternatives for managing extensive epidermal damage.

## 4. Discussion

This study highlights the potential of PLA-based membranes as an adjunctive therapy for bullous-impetigo-associated skin erosions that go beyond the traditional antibiotic management. In the cases presented, the application of Suprathel^®^ in combination with oral antibiotics resulted in accelerated wound closure, with complete re-epithelialization occurring within 7 to 14 days—a significantly faster recovery compared to the historical averages for bullous impetigo [6]. Notably, pain control was achieved rapidly, with complete pain relief by days 2–3, a key advantage not documented in prior studies on impetigo management [1,2,5,6,17].

For localized bullous impetigo, the standard first-line treatment consists of topical antibiotics, such as mupirocin, retapamulin, and ozenoxacin, which reduce the bacterial burden and shorten the disease duration [1,5,7,17,18]. However, the treatment duration varies by product, and the lesion extent can lead to heterogeneous distributions of antibiotics, limiting their efficacy. Chlorhexidine rinses and diluted bleach baths are often employed as adjuncts to improve bacterial decolonization, but their effectiveness depends heavily on their proper application and clinician expertise [2,5,7,18]. In contrast, the PLA membrane provides a uniform wound interface, ensuring continuous antibacterial effects and promoting faster epithelialization [8,16].

The mechanism of action of Suprathel^®^ in wound healing is largely attributed to the controlled release of lactate as the membrane gradually degrades within the wound bed [19,20,21,22]. Lactate is a key bioactive metabolite that enhances angiogenesis, supports cellular proliferation and survival, and promotes extracellular matrix (ECM) deposition [15,19]. Additionally, lactate plays a crucial role in modulating the inflammatory response, reducing excessive immune activation while supporting wound resolution [23,24,25]. The acidification of the wound environment resulting from lactate metabolism further contributes to wound healing by lowering the pH, which exerts two critical effects. First, a reduction in pH enhances the unloading of oxygen from hemoglobin via the Bohr effect, increasing the tissue availability of oxygen in the wound bed [26]. Second, a mildly acidic environment inhibits bacterial proliferation, as most pathogenic bacteria, including *S. aureus*, exhibit reduced virulence in acidic conditions [16]. These combined effects explain the rapid wound closure observed in our cases and suggest that PLA membranes provide a multifaceted therapeutic advantage beyond passive wound coverage.

Pain control was another significant outcome of this study. This analgesic effect can be attributed to two primary mechanisms. First, the PLA membrane acts as a physical barrier, shielding exposed nerve endings from mechanical stimuli, thereby reducing nociceptor activation. Second, lactate has been identified as a potent inhibitor of the transient receptor potential vanilloid-1 (TRPV-1) pain channel, which is involved in nociceptive signaling and inflammatory pain [19]. This dual mechanism explains both the immediate relief observed post-application and the progressive deepening of analgesia over time as lactate release continues. This finding is in line with evidence from burn treatment studies that have demonstrated that Suprathel^®^ induces a statistically significant reduction in pain compared to that with any other standard-of-care therapy [9].

Our findings align with previous studies on PLA-based alloplastic skin substitutes, particularly in the context of burn treatment. Initially, PLA membranes were introduced for partial-thickness burns, especially in pediatric populations, where they were associated with significantly reduced exudation, improved re-epithelialization, superior scar quality, and reduced pain and need for opioid medication [20,21,22]. More recent research has demonstrated their potential role in managing chronic wounds at risk of infection [27,28]. Consistent with these observations, our study found complete wound closure and the normal appearance of the skin at 30 days, with no residual erythema, scarring, or post-inflammatory hyperpigmentation.

Despite these promising results, we acknowledge the limitations of this study. It is a small retrospective case series, and although impetigo is a common dermatological condition, extensive bullous involvement is rare. While our findings suggest that PLA-based membranes provide a viable alternative to conventional wound dressings, additional cases are needed to establish the consistency of these outcomes further. Furthermore, we recognize the need for comparative studies to determine whether Suprathel^®^ significantly reduces healing times compared to those under standard care. Prospective, controlled trials evaluating PLA membranes’ efficacy for different severities of impetigo would provide stronger evidence for its widespread adoption in dermatological wound management.

## 5. Conclusions

Our findings support the use of PLA-based synthetic skin substitutes as a safe and effective treatment modality for bullous-impetigo-associated skin erosions. The ability of PLA membranes to accelerate wound healing, modulate the inflammatory environment, reduce the bacterial burden, and provide superior pain relief makes them a compelling adjunctive therapy in cutaneous infections.

Given the increasing challenges of antibiotic resistance and the limitations of conventional therapies, adopting bioresorbable synthetic membranes represents a paradigm shift in managing infectious skin conditions. Our findings support adopting this technology in cases where the traditional approaches may be suboptimal, paving the way for a more effective, patient-centered, and resource-efficient treatment strategy for impetigo and other superficial bacterial skin infections.

## Figures and Tables

**Table 1:** Demographic table of the patients studied. TMP-SMX: Trimethoprim–Sulfamethoxazole.

Patient	Sex	Age	Comorbidities	Therapeutic Regimen	Adjunct Treatment	Outcome	Time of Healing After Application of Suprathel^®^
1	Female	61	High blood pressure, diabetes mellitus type 2	Piperacillin–Tazobactam (7 d) and amoxicillin (14 d)	Suprathel^®^	Fully closed by day 7, no recurrence on 4-week follow-up	7 days
2	Female	50	No comorbidities	TMP-SMX (7 days)	Suprathel^®^	Fully closed by day 14, no recurrence on 4-week follow-up	14 days
3	Female	90	High blood pressure, diabetes mellitus type 2, chronic renal failure	TMP-SMX (7 days)	Suprathel^®^	Fully closed by day 7, no recurrence on 4-week follow-up	7 days
4	Female	82	Diabetes mellitus type 2	Amoxicillin–clavulanate, TMP-SMX (7 d)	Topical mupirocin	No closure at 12-week follow-up, multiple recurrences	N/A
5	Female	62	Diabetes mellitus type 2	Amoxicillin–clavulanate, TMP-SMX (7 d)	Topical mupirocin	Partially closed by day 21, multiple recurrences and hyperkeratosis on 12-week follow-up	N/A
6	Female	60	No comorbidities	Amoxicillin–clavulanate, TMP-SMX (7 d)	Topical mupirocin	Partially closed by day 21, recurrence and hyperkeratosis on 12-week follow-up	N/A
7	Female	69	Diabetes mellitus type 2, chronic venous insufficiency, history of heavy smoking	Amoxicillin–clavulanate, TMP-SMX (7 d)	Topical mupirocin	No closure at 12-week follow-up, superinfection with *E. coli*	N/A
8	Male	71	Diabetes mellitus type 2, chronic venous insufficiency, active heavy smoking	Amoxicillin–clavulanate, TMP-SMX (7 d)	Topical mupirocin	Partially closed by day 21, recurrence, hyperkeratosis and severe edema on 12-week follow-up	N/A

## Data Availability

No new data was created or analyzed in this study. The original contributions presented in this study are included in the article. Further inquiries can be directed to the corresponding author.

## Abbreviations

The following abbreviations are used in this manuscript:

MRSA	Methicillin-resistant *Staphylococcus aureus*
PLA	Polylactic acid
TMP-SMX	Trimethoprim–Sulfamethoxazole
